# New insights at the interface between leprosy and immune-mediated rheumatic diseases

**DOI:** 10.3389/fmed.2023.1239775

**Published:** 2023-09-25

**Authors:** Vitor Alves Cruz, Cleandro Pires de Albuquerque, Maria Fernanda Brandão de Resende Guimarães, Carla da Fontoura Dionello, Sandra Lúcia Euzébio Ribeiro, Viviane Angelina de Souza, Ciro Martins Gomes, Licia Maria Henrique da Mota

**Affiliations:** ^1^Federal University of Goiás, Goiás, Brazil; ^2^Department of Rheumatology, University of Brasilia, Brasilia, Brazil; ^3^Department of Rheumatology, Federal University of Minas Gerais, Belo Horizonte, Brazil; ^4^Department of Rheumatology, Federal University of Rio de Janeiro, Rio de Janeiro, Brazil; ^5^Department of Rheumatology, Federal University of Amazonas, Manaus, Brazil; ^6^Department of Rheumatology, Federal University of Juiz de Fora, Juiz de Fora, Brazil; ^7^Department of Dermatology, University of Brasilia, Brasilia, Brazil

**Keywords:** rheumatic dieases, leprosy, SLE, rheumatoid arthritis, *Mycobacterium leprae*

## Abstract

Leprosy is an infectious and contagious disease of slow evolution, triggered by *Mycobacterium leprae*. Arthritis is its third most common manifestation, after cutaneous and peripheral nerve involvement. Since musculoskeletal symptoms may be the initial presentation of the disease, it is important for health professionals to recognize its rheumatic manifestations for early diagnosis and appropriate treatment, especially in endemic areas. In addition, cases of leprosy have increased globally, notably in patients undergoing treatment with TNF-α blockers and due to the increase in migration and travel of people from developing countries to developed countries. This review proposes to discuss the main scenarios of mimicry of different rheumatic diseases by leprosy, as well as the role of immunosuppressive drugs used in rheumatology practice in the treatment of reactional states and in the risk of developing the infection.

## 1. Introduction

Leprosy is a stigmatizing and discriminating chronic infectious disease that has been cited in the literature of ancient civilizations until today. The disease is caused by *Mycobacterium leprae* and *Mycobacterium lepromatosis* and affects most commonly the skin and peripheral nerves but may evolve systemically mimetizing other inflammatory diseases, such as the rheumatic, with many different clinical manifestations (1).

Leprosy is still considered a highly neglected tropical disease (NTD), with more than 200,000 new cases reported every year (2). Despite efforts to eliminate leprosy as a public health problem, it remains prevalent in many regions of the world, especially in Brazil, India, and Indonesia. The bacilli are probably transmitted through the respiratory tract, and those in close and prolonged contact with someone with untreated leprosy are at major risk of acquiring the disease. Most people have a natural immunity to *M. leprae*, which means they are unlikely to contract the disease even if they encounter an infected person. The incubation period of leprosy is typically between 2 and 5 years, although it can range from 6 months to 20 years (2).

Several risk factors have been identified for developing leprosy. These include living in areas with a high burden of the disease, having close contact with an infected individual, and having a weakened immune system due to other health conditions such as HIV/AIDS and medication-induced immunosuppression. Poverty, poor nutrition, and inadequate healthcare also increase the risk of developing leprosy (3, 4).

Genome-wide association studies (GWAS) enabled the identification of genes involved in the innate and adaptive immune responses potentially associated with increased susceptibility to leprosy, with an impact on determining the phenotype of the disease. They are related to key cells in the pathogenesis of leprosy, such as macrophages (VDR, OPA1, SLC7A2, and RAB32), dendritic cells (TLR1, TLR2, NOD2, MICA, and MICB), keratinocytes (FLG), and T lymphocytes (IL23R, IL12B, TNFSF15, TYK2, and SOCS1) (5).

Early diagnosis and treatment of leprosy are important to break the transmission chain and prevent physical disabilities. Bazan-Furini et al. followed 320 household contacts of leprosy patients and concluded that anti-PGL-1 positivity could be used to identify patients at higher risk of developing leprosy (6). Santos et al. in another Brazilian study, analyzed 361 contacts with positive anti-PGL-1 that were asymptomatic, identifying the mycobacterium in the PCR test of the skin biopsy in 35% of the cases and alterations in the electroneumyography in 23.5%, concluding that screening with anti-PGL-1 can be useful to identify latent cases of leprosy (7).

A leprosy case is defined by the detection of, at least, one of the following cardinal signs: (1) definite loss of sensation in a pale (hypopigmented) or reddish skin patch; (2) thickened or enlarged peripheral nerve, with loss of sensation and/or weakness of the muscles supplied by that nerve; and (3) microscopic detection of bacilli in a slit-skin smear (3).

Leprosy is characterized by two stable polar forms with diverse immunopathological and clinical aspects. In the tuberculoid (TT) pole, the host presents an efficient immune response mediated by cells against *M. leprae*. At the other pole, the lepromatous (LL) form is characterized by an inefficient immune response mediated by cells, with great bacillary multiplication and dissemination of the disease. Between these two extremes, there are intermediate forms, dimorphous, which reflect gradual variations in resistance to the bacillus (2, 3).

During the course of the disease, acute exacerbations, defined as leprosy reactions, may occur. They are classified into type 1 (T1R), or reversal reaction, and type 2 (T2R), or erythema *nodosum* leprosum reaction. T1R often occurs in borderline form and is associated with increased cell-mediated immune response against the mycobacteria, characterized by a Th1-type immune response (IFN-γ, TNF-α, and IL-2), by CD4^+^ T cells in the skin and nerves, joints, and other tissues. T2R occurs predominantly in patients with the lepromatous clinical form, with high circulating levels of TNF-α, infiltration of neutrophils, and activation of the complement cascade leading to an intense inflammatory response (3).

The mechanisms of arthritis in leprosy are not fully understood. There are cytokines involved in both the Th1 and Th2 responses, including TNF-α, IL-6, IL-8, and IL-10. It is believed that reactional states (types I and II), direct infiltration of the synovium, and peripheral sensory neuropathy are the main determinants of joint involvement (4).

The differential diagnosis between leprosy and immune-mediated rheumatic diseases (IMRD) can represent a great challenge since the infection can reproduce not only the clinical manifestations but also the laboratory findings of these conditions. This review proposes to discuss the main scenarios of mimicry of different IMRD by leprosy, as well as the role of immunosuppressive drugs used in rheumatology practice in the treatment of reactional states and in the risk of developing the infection.

## 2. Rheumatic diseases mimicked by leprosy

### 2.1. A challenge in the differential diagnosis

#### 2.1.1. Rheumatoid arthritis

Articular and periarticular manifestations in all forms of leprosy are already well described in the literature and are considered the third most frequent manifestation after dermatological and neurological involvement. In rheumatological practice, it can present as primary arthritis or even as an infection complicating immunosuppressive therapy for a chronic immune-mediated inflammatory disease. It should always be considered among the differential diagnoses of arthritis in endemic regions (4, 8).

The spectrum of musculoskeletal manifestations related to leprosy is quite varied (Table 1), including Charcot arthropathy secondary to peripheral sensory neuropathy, swollen hands and feet syndrome, septic arthritis, acute polyarthritis of the leprosy reaction, and chronic arthritis caused by infiltration directly from the synovium by the leprosy bacillus (9).

**Table 1:** Clinical and joint features in leprosy.

Clinical presentation	Onset	Symmetry	Poliarthritis	Joints involved	Erosions	*M. leprae* in the synovium
Leprosy reaction	Acute	Yes	Yes	Wrist, MCP, MTP, PIP, and ankle	Yes/no	Yes
Hands/feet syndrome	Acute	Yes	Yes	Ankle, wrist, PIP, and MTP	No	Yes
Chronic arthritis	Insidious	Yes	Yes	Wrist, MCP, MTP, PIP, and knee	Yes	Yes/no
Charcot arthropathy	Insidious	No	Mono or Polyarticular	Hands and feet, ankle, knee, and wrist	Yes	No
Tenosynovitis	Insidius or acute	Yes/No	No	Extensor tendons of hands, and feet	No	Yes, in the synovial sheath of the tendons

Modified from Chauhan et al. (8). PIP, proximal interphalangeal; MCP, metacarpophalangeal; MTP, metatarsophalangeal.

It is not clear whether arthritis in these patients would be due to the presence of the intra-articular bacillus, such as infectious arthritis, or whether it would be an immune response triggered by *M. leprae* antigens that could lead to the production of circulating immune complexes with complement consumption resulting in vasculitis and inflammation, or a reaction to bacterial antigens, similar to reactive arthritis (10). Holla et al. performed synovial biopsy in 36 patients with lepromatous leprosy and knee arthritis not associated with reactional states. The synovial lining showed villous hyperplasia and hypertrophy, congestion, pannus formation, granulomas containing macrophages, and intact bacilli in nine patients (11).

**Figure 1:**
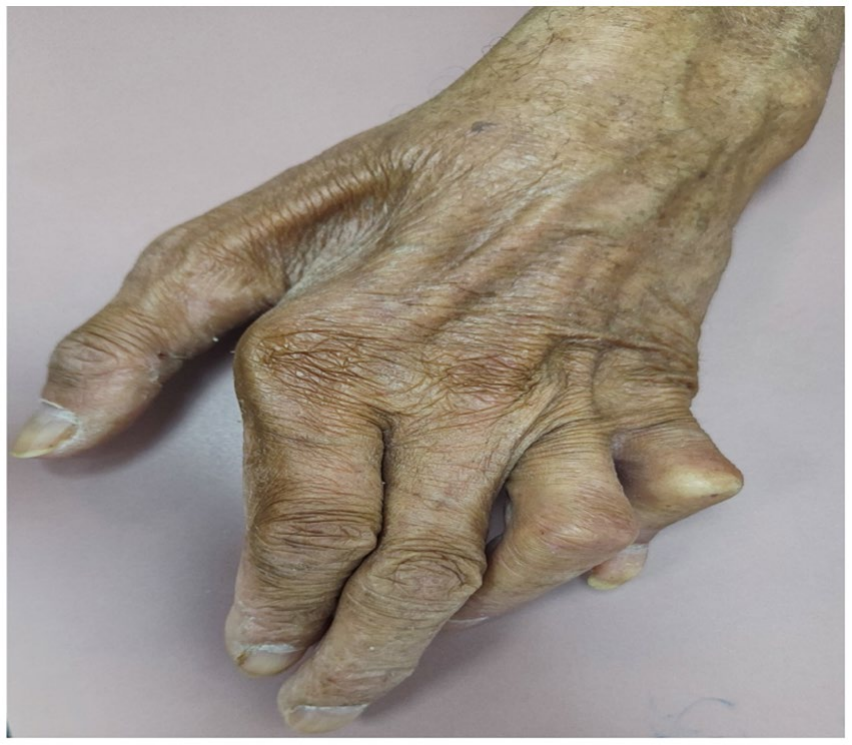
Brazilian male patient, diagnosed with leprosy, presenting with polyarthritis and deformities.

Chronic symmetrical polyarthritis (Figure 1), symmetrically affecting the wrists, hands, feet, and ankles, similar to rheumatoid arthritis (RA), can occur without any evidence of reactional states, affecting both genders equally. Its typical symptoms are joint pain and swelling, associated with joint effusion, periods of exacerbation and remission, and morning stiffness in 30% of the patients. Late diagnosis often leads to deformities such as boutonniere, swan neck, and hammer toes associated with ulnar deviation. Leprosy-related arthritis has morning stiffness of a shorter duration, the absence of extra-articular manifestations of RA, a negative rheumatoid factor in most patients, and less erosive radiological findings (12). Two Indian studies observed the prevalence rates for arthritis in leprosy at 61.4 and 10%, respectively (13, 14).

The most common radiological alterations are fusiform edema of the periarticular soft tissues, bone porosity, eventual periarticular erosions, and narrowing of the joint space. The detection of low levels of autoantibodies such as rheumatoid factor and anti-cyclic citrullinated peptide antibodies may occur and represent a problem in the differential diagnosis. In a Brazilian study, Dionello et al. demonstrated that anti-CCP antibodies and rheumatoid factor were detected in leprosy patients in 9.3 and 41%, respectively (15).

Cossermelli-Messina et al. described 39 cases of leprosy with chronic arthritis of the RA type, with an average duration of 11 years. Although these patients experienced considerable relief with anti-leprosy therapy, the arthritis never fully resolved and was complicated by neuropathic features (16).

The absence of rheumatoid nodules, specific autoantibodies, and responsiveness to anti-leprosy treatment are important characteristics that help in the recognition of chronic arthritis associated with leprosy and in its differentiation from RA. In addition, the radiographic changes are generally less pronounced in relation to RA (17, 18).

In patients with exuberant cutaneous manifestations, bacilloscopy and biopsy of the lesions are decisive in the diagnostic process. In cases where arthritis is an isolated manifestation, a synovial biopsy can be useful in the differential diagnosis. Polymerase chain reaction (PCR) can aid in defining the leprosy diagnosis in suspected patients with clinically suggestive or atypical lesions presenting with negative baciloscopy and inconclusive histopathology. Choosing the right genetic target leads to an important improvement in sensitivity and specificity through the identification of repetitive genetic sequences, such as a specific repetitive element—RLEP, an assay considered highly specific for *M. leprae* and capable of detecting a small amount of DNA or approximately 300 bacteria in infected tissues (19). Nohanty et al. in their study with 80 patients, observed a greater sensitivity of RLEP-PCR compared to bacilloscopy (66 × 22%), highlighting the importance of the method in the context of the most challenging differential diagnoses (20).

Although the treatment of chronic arthritis, mainly with the use of corticosteroids, can slow the progression of neuronal damage in patients with subclinical neuritis, there is also the possibility that permanent joint damage will exacerbate the functional limitations caused by neuropathy related to the disease or the use of thalidomide. Treatment includes the use of analgesics, non-steroidal anti-inflammatories, and corticosteroids. However, the fundamental principle is the use of antimicrobials through multi-drug therapy (17, 18).

## 3. Systemic lupus erythematosus

Systemic lupus erythematosus (SLE) is an autoimmune disease with multisystem involvement. Primary dysfunction of the innate and adaptive immune systems contributes to the uncontrolled production of autoantibodies and their specific clinical manifestations. In addition, the immune derangements also increase susceptibility to infections, and microorganisms may act as triggers for disease reactivation (21).

In leprosy infection, after bacilli uptake, dendritic cells modulate inflammation with the production of cytokines and chemokines and regulate adaptive cell-mediated immunity into a Th1 or Th2 response. The ability of *M. leprae* to regulate cytokine production and to drive Th1 or Th2 responses may contribute to clinical presentation. These immune abnormalities are also responsible for acute reactions that may occur at any moment during the infection: TR1, reversal reaction, and TR2, erythema nodosum leprosum. Although the mechanisms of these reactions are not well understood, many of the clinical manifestations may mimic rheumatic diseases (16). Ribeiro et al. recently suggested that vitamin D deficiency might have a pathogenic link to the emergence of autoimmunity in patients with leprosy (22).

Leprosy may mask a variety of diseases, and its early diagnosis is of the utmost importance to institute appropriate treatment and reduce patient morbidity and mortality (23).

Frequently, leprosy patients exhibit symmetrical polyarthritis, lymphadenopathy, serositis, necrotizing vasculitis, hemolytic anemia, alopecia, photosensitivity, glomerulonephritis, and many skin lesions, including the rare and severe Lucio’s phenomenon that leads to purpura fulminans. All of these manifestations could mimic rheumatic diseases, including SLE (21, 23).

Similar to leprosy, skin manifestations in SLE can be heterogeneous. The similarities between infiltrated plaques in borderline leprosy and lupus tumidus may represent a diagnostic challenge. The lack of a high index of suspicion can lead to a misdiagnosis (18). Teixeira et al. reported a 9% frequency of discoid lesions in leprosy patients, which may be explained by misdiagnosis. Although its clinical presentation may be similar to that of chronic cutaneous lupus erythematosus, certain features of leprosy should alert the physician, such as anesthetic skin lesions, nerve enlargement, and nerve tenderness. Lesions may affect cutaneous peripheral nerves, primarily the posterior tibial nerve, ulnar, median, and lateral popliteal (24).

A Brazilian study demonstrated a high prevalence of some of the SLE criteria in one hundred lepromatous leprosy (LL) patients. The criteria with the highest prevalence were malar erythema (44%), arthritis (23%), photosensitivity (29%), lymphopenia (19%), and the presence of antiphospholipid antibodies, including immunological criteria (20%). The specificity found (84%) was lower than the specificity allocated to the criteria in 1997 by the ACR. The authors concluded that leprosy in multi-bacillary forms mimics the clinical and laboratory characteristics of SLE, and thus physicians need to be aware of the realities of local infectious diseases before affirming a definitive diagnosis of SLE (24).

Besides mimicking SLE, leprosy can also induce lupus flares. One study presented three Brazilian patients with longstanding quiescent SLE who had a concomitant diagnosis of leprosy and developed new lupus manifestations. These included polyarthritis, systemic symptoms, cutaneous lesions, hemolytic anemia, and lupus nephritis (21).

Another possible association would be the diagnosis of leprosy mimicking SLE. An Indian study described a case diagnosed as leprosy that subsequently evolved to a diagnosis of SLE with severe clinical manifestations, including pulmonary hemorrhage. The authors discussed if this patient had lupus since the beginning of the clinical manifestations, and lupus autoimmunity was moderately controlled with dapsone and clofazimine. However, vice versa might also be true since cases of lupus may be diagnosed as leprosy due to the immunological features and overlap (25).

Leprosy can also induce the expression of many autoantibodies. The multi-bacillary status may exert a potent trigger for immune complex production. Antinuclear antibody (ANA) positivity may be related to weak cross-reactivity between mycobacterial antigens and human DNA associated with continuous stimulation of B cells due to cell destruction (1). Other pathogenic mechanisms include molecular mimicry, and some studies reported shared idiotypes among antibodies derived from patients with leprosy and SLE (26–30).

Antinuclear antibody (ANA) positivity may reach 30%, and one study described a similar prevalence of anti-ssDNA antibodies between SLE and leprosy patients. ANA is usually present at a low titer and with speckled and homogenous patterns. However, cases of leprosy without the coexistence of SLE with high titers have also been reported (21, 23). A Brazilian study showed that more specific autoantibodies, such as anti-dsDNA, anti-SM, and anti-P, are infrequently found in leprosy sera (28).

Given the clinical and laboratory similarities between the diseases, in patients with suspected SLE, especially in endemic areas, even in scenarios where the classification criteria are fulfilled, leprosy must be considered in the differential diagnosis.

## 4. Systemic sclerosis

Systemic sclerosis is one of the diseases that leprosy can mimic. Skin thickening may occur due to the edema of leprosy plaques. Other clinical manifestations include Raynaud’s syndrome, telangiectasias, and resorption of the distal phalanges. Resorption of the distal phalanges occurs in 20–25% of patients with systemic sclerosis and is strongly associated with severe digital ischemia, suggesting that it is due to ischemic atrophy. In leprosy, alterations of the distal phalanges can be of various types, occurring in 19–45% of patients (31).

Bone resorption thins and/or shortens the phalanges, metacarpals, and metatarsals. The distal resorption decreases bone length, while the reabsorption of trabecular bone, also called concentric bone atrophy, decreases width. The combination of both gives the bone an appearance called licked candy stick. In the hands, bone resorption starts in the extremities of the distal phalanges, sites most subject to trauma, with subsequent involvement of the middle and proximal phalanges and, more rarely, the metacarpal bones. In the presence of associated secondary infections, the process of resorption progresses more quickly and may cause the loss of digits (31).

Chu et al. described a case of leprosy mimicking systemic sclerosis. The patient presented with sclerodactyly and Raynaud’s syndrome, no history of digital ulcerations, and progressive reabsorption of the distal phalanges. He also had symptoms of sensory polyneuropathy in the hands and feet and telangiectasia on the face. There was no pulmonary or esophageal involvement (32).

### 4.1. Myositis

The skeletal muscles may be involved in leprosy because of peripheral neuropathy and muscular denervation or as a primary muscle disease with an inflammatory reaction, which is referred to as lepromatous myositis. The muscle involvement in leprosy may also be due to the systemic spread of bacilli. Subclinical muscle involvement may occur, and myonecrosis and myophagocytosis are rare manifestations. The exact incidence of leprosy myositis is not known; this manifestation is usually asymptomatic, with descriptions of its occurrence in 45–60% of leprosy cases (33).

The muscle biopsy may show variation in fiber size, type 1 and/or type 2 fiber atrophy, type 1 fiber predominance, and grouping. These changes are consequent to mononeuropathy, multiplex neuropathy, or polyneuropathy. Inflammation is prominent in the perimysium and may extend into the muscle along the interstitial connective tissue and involve the nerve twigs. Granulomas and acid-fast bacilli may be detected, especially in LL (34).

Three stages may develop: an initial stage of invasion and proliferation of *M. leprae* inside muscle fibers, followed by muscle fiber degeneration and infiltration by polymorphonuclear leukocytes, lymphocytes, macrophages, and the bacilli fragmentation, and finally, muscle fiber destruction, fibrous tissue replacement, vacuolation of the macrophages, and complete disappearance of the bacilli (34).

Albert et al. described a case of a patient with a skin rash and muscle weakness that simulated the appearance of dermatomyositis. However, electroneuromyography did not document muscle involvement. The skin lesions revealed hypoesthesia despite their similarity to Gottron papules. They observed three additional leprosy patients with elevated CPK levels, suggesting that elevated muscle enzymes may be a common manifestation and that the rheumatologist must be alert to cases of inflammatory myopathy that do not respond adequately to immunosuppression (35).

Inflammatory myopathies, among other IMRDs, and leprosy share in their pathophysiology alterations in the expression of microRNAs (miRNAs). miRNAs are small, non-coding RNAs capable of regulating gene expression through the degradation or repression of translation of target messenger RNA molecules. They seem to play a major role in demyelination and neuropathic pain in leprosy (36, 37).

## 5. Spondyloarthritis

Other rheumatological manifestations can be observed in leprosy. Among them, it is important to mention the joint manifestations that can mimic spondyloarthritis (SPA), presenting as an asymmetric oligoarthritis of the lower limbs (37, 38). A retrospective study carried out in India analyzed patients who were referred to the unit of rheumatology and were later diagnosed as having leprosy. Arthritis was found in 50% (*n* = 22) of patients at presentation, and one-third of those with arthritic manifestations had asymmetric lower limb oligoarthritis like the pattern observed in SPA (37). Another study, in Brazil, analyzed 55 patients with leprosy-related arthritis and identified the oligoarticular pattern in 20 (36.3%). The most frequently affected joints were the wrists and the ankles (12). In a third study that evaluated a population of pediatric patients with leprosy in Brazil, at least one musculoskeletal manifestation (arthralgia, arthritis, and/or myalgia) was observed in 14% of patients with leprosy (*n* = 50), and five had asymmetric joint involvement (39).

Enthesitis is another described condition, and a study of 77 patients across the leprosy spectrum showed 10 patients with enthesitis, which had not been previously described to the best of our knowledge and was not associated with the reaction characteristics of erythema nodosum leprosy (40). The manifestation of enthesopathy seems to appear late in the course of the disease. The mechanism involved is probably recurrent local microtrauma of zones previously affected by inflammation, and a contributory factor can be the altered proprioceptive sensitivity in the late leprosy stage (41).

Sacroiliitis is not common, although the presence of sclerosis, erosions, and narrowing of the cartilage space in the sacroiliac joints was present in 35 of 55 radiographs in a study. It did not correlate with lower back pain, and LL was the most common subtype associated with this manifestation (9).

Dactylitis, a common presentation in psoriatic arthritis, was recently reported in a 32-year-old woman diagnosed with a leprosy reaction—erythema nodosum leprosy. In addition to dactylitis, she had a 1-month history of recurrent painful nodules on the legs and forearms, bilateral symmetric polyarthritis of small and large joints, and swelling of the dorsum of the hands and feet (42).

In conclusion, rheumatic manifestations that simulate spondyloarthritis can be observed in leprosy patients. The appearance of asymmetrical oligoarthritis is often observed. Other less common manifestations already reported in the literature are enthesopathy, sacroiliitis, and dactylitis.

## 6. Fibromyalgia

Leprosy causes significant pain in affected patients, especially in those with pure neural forms. Fibromyalgia (FM) is characterized by diffuse pain, fatigue, unrefreshing sleep, memory disturbances, anxiety, and depression. It can be confused with leprosy, which is also characterized by pain as an initial symptom, especially in patients without skin lesions (43).

Alterations in the sensitivity of peripheral nerves should be investigated in patients with suspected FM, especially in regions where leprosy is endemic. This is the most important clue for the differential diagnosis. Both diseases can occur simultaneously, with overlapping or mimicking signs and symptoms (44).

Pure neural leprosy often results in diffuse pain, which can mimic the FM presentation. It affects about 3–10% of patients. The diagnosis is based mainly on electrophysiological studies, peripheral nerve biopsy, molecular tests, and the dosage of anti-phenolic glycolipid antibody (anti-PGL-1). Nerve conduction studies as part of electroneuromyography (ENMG) may reveal a mononeuropathy, a mononeuropathy multiplex, or even a mononeuropathy multiplex confluent (45). Patil et al. observed in their study the presence of immunocomplexes in 87.5% of patients with neuritis, suggesting an important role for this event in the neuronal damage associated with the disease (46).

A systematic and regular assessment of the peripheral nerves is an essential step in leprosy control. The neurological examination enables early diagnosis and treatment of neuritis, as well as monitoring their evolution, and should be prioritized by health professionals, providing objective subsidies for determining conduct, especially in pure neural forms (45, 46).

## 7. Antiphospholipid syndrome

Antiphospholipid antibodies (aPLs) make up a large group of antibodies responsible for the pathophysiology of arterial or venous thrombotic conditions, in addition to recurrent miscarriages, known as antiphospholipid syndrome (APS). This group includes anti-cardiolipin (aCL), anti-β2-GPI, and lupus anticoagulant (LA) antibodies. The presence of these antibodies in patients with leprosy may indicate a risk factor for the development of leprosy reactions or even the Lucio phenomenon, characterized as an exacerbated vasculitis (47).

Ribeiro et al., in their study with 158 patients, observed a high prevalence of aCL and anti-β2-GPI predominantly IgM, some in high titers, in patients with leprosy particularly in the multibacillary forms, with a significant difference when compared with healthy controls. Five years later the patients were re-evaluated and none developed thrombotic phenomena (26). De Larrañaga et al. evaluated 51 leprosy patients without any clinical characteristic of APS and identified positive AL in 35 patients, aCl in 31, and anti-β2GPI antibodies in 29 patients, with no statistical difference between the multi- and pauci-bacillary forms. Although the presence of aPL is not necessarily associated with the development of APS, it can make the differential diagnosis difficult in cases of vasculitis secondary to leprosy (48).

Another possible confounding factor is the thromboembolic phenomenon related to the use of thalidomide. In the last decade, some case reports have described the occurrence of thromboembolism related to its use in the treatment of type II leprosy reaction. It is unclear whether the high frequency of antiphospholipid antibodies could impact the increased prevalence of these events (49).

Differential diagnosis between Lucio phenomenon and antiphospholipid syndrome is particularly difficult, as patients with leprosy exhibit a high prevalence of antiphospholipid antibodies (predominantly IgM isotype), most frequently in lepromatous forms, regardless of disease duration or treatment exposure. Guevara et al. reported a case of a 32-year-old woman who presented with a sudden onset of fever and skin necrosis on the lower part of the legs. She was treated for APS due to the presence of antiphospholipid antibodies but had an inadequate response. Skin biopsy revealed thrombotic vasculopathy and necrotizing vasculitis associated with foam cell aggregation in the perivascular and subcutaneous areas, with acid-fast bacilli in histiocytes and blood vessel walls. The pathology confirmed the diagnosis of Lucio’s phenomenon, and with adequate antimicrobial therapy, there was an improvement in the condition (50).

In summary, the differential diagnosis between APS and leprosy should always be carried out considering that both conditions cause skin lesions, either due to thrombotic or neuropathic origin, and APL positivity is frequently observed in both situations.

## 8. Cutaneous and systemic vasculitis

Vasculitis is a well-known, relatively uncommon histological finding in erythema nodosum leprosum (51, 52). Moreover, leprosy can mimic various forms of cutaneous and systemic vasculitis. Features such as skin nodules, necrotic ulcers, purpura, arthritis/arthralgia, peripheral neuropathy, renal involvement, fever, elevated inflammatory markers, and circulating autoantibodies—all reported in leprosy—represent a challenge in differentiating leprosy from immune-mediated rheumatic diseases and primary vasculitis (such as polyarteritis nodosa), usually at the cost of delayed diagnoses (53, 54).

In leprosy-associated vasculitis, bacterial lipopolysaccharides induce the secretion of TNF and IL1 by activated macrophages, which stimulate endothelial cells to produce prostaglandins, IL6, and factor III, with formation of thrombi in the capillaries (55).

A severe inflammatory-thrombotic vasculopathy that rarely occurs in leprosy is called the Lucio phenomenon (LP). LP is an acute, life-threatening, necrotizing skin reaction usually observed in untreated patients with diffuse lepromatous forms with high bacillary loads. In most reports, LP occurred as the first overt manifestation of leprosy in patients previously undiagnosed; in a minority of cases (<15%), LP occurred during the treatment or even after its completion (56, 57). Clinically, LP is characterized by outbreaks of painful, sharply delineated erythematous-purpuric macules with central necrosis and subsequent irregular skin ulceration in limbs (mainly), trunk, and face, leaving stellar scars; the skin is diffusely infiltrated; and up to 50% of the patients have fever.

Considering the rarity of vasculitis and the difficulty of its differential diagnosis, a tissue biopsy should be performed in all cases, maintaining high clinical suspicion in endemic areas (58). The histopathology reveals a large number of acid-fast bacilli (AFB) in the endothelium, which is proliferated with areas of fibrinoid necrosis, vascular thrombotic occlusion with ischemic epidermic necrosis, discrete lympho-histiocytic inflammatory infiltrates, abundant AFB in macrophages that infiltrate the walls of skin vessels (along with AFB themselves), and leukocytoclastic vasculitis (56, 57). Anemia, leukocytosis, and elevated acute phase reactants are common. Mortality is high (>one-third), usually due to sepsis and infectious complications. Treatment usually involves multi-drug anti-leprosy therapy, steroids, anticoagulants, systemic antibiotics, surgical debridement, and wound care.

Apart from LP, data on leprosy as a mimic of other vasculitis syndromes are scarce, largely based on case reports. Ribeiro et al. described the cases of two women with multi-bacillary leprosy, exhibiting erythematous-violaceous ulcerated skin lesions, livedo reticularis, polyarthralgia, myalgia, and edema of the lower limbs, mimicking systemic lupus erythematosus and polyarteritis nodosa (59). Sampaio et al. report the case of an 86-year-old woman with polyarthritis, subcutaneous nodules, and leg ulcers initially suspected of primary vasculitis and eventually diagnosed with leprosy (60).

Yu et al. presented the case of a 26-year-old male patient with high fever (39.5°C), ulcerating cutaneous nodules, numbness of limbs, coughing, myalgia, arthralgia, lymphadenomegaly, leukocytosis, elevated inflammatory markers, and positive ANCA/anti-myeloperoxidase, initially diagnosed as ANCA-associated vasculitis and treated with methylprednisolone and cyclophosphamide, but eventually revealing multi-bacillary leprosy on biopsy studies, with resolution of symptoms following multi-drug therapy (61). Anti-neutrophil cytoplasmic antibodies with both perinuclear and cytoplasmic patterns (p- and c-ANCA), as well as atypical patterns, have been observed by indirect immunofluorescence in patients with lepromatous and borderline leprosy (62, 63).

Baharuddin et al. reported on a 70-year-old male patient with an 8-week history of neuropathic complaints, ulcerated skin lesions on fingers, elbow bursitis, tenosynovitis, and elevated inflammatory markers, resembling a rheumatic disease with cutaneous vasculitis. A skin and bursa biopsy revealed paucibacillary leprosy, showing excellent response to multi-drug therapy (64). Bowen et al. described the case of a 46-year-old male patient who developed multifocal, sensitive peripheral neuropathy 25 years after completing leprosy treatment. A nerve biopsy revealed vasculitis without AFB; immunohistochemistry showed deposits of mycobacteria antigens within nerves and vessel walls (65).

Manoj et al. reported the case of a 38-year-old woman with 3 years of symmetrical polyarthritis and swan-neck deformities and a few months of ocular pain with diminution of vision and paresthesia in hands and feet. Inflammatory biomarkers were elevated, and rheumatoid factor was positive (low titer). Nerve conduction studies detected mononeuritis multiplex, and an ophthalmologist confirmed scleromalacia perforans. Rheumatoid arthritis and vasculitis (mononeuritis) were diagnosed, and the patient received pulse methylprednisolone, planning for subsequent cyclophosphamide. Meanwhile, a sural nerve biopsy disclosed multi-bacillary leprosy. Multi-drug therapy led to the resolution of the inflammatory symptoms, including arthritis (66). Bhattacharjee et al. reported on a young adult male with 2 months of fever, arthralgia, hypoesthesia in hands and feet, and multiple tender, ulcerative skin nodules on the trunk and extremities, mimicking cutaneous vasculitis. A slit-skin smear showed multiple AFB, and histopathology of the nodules confirmed necrotic erythema nodosum leprosum (ENL) (67).

## 9. Role of synthetics conventional disease-modifying antirheumatic drugs and immunosupressive agents in the treatment of leprosy reactions

Patients with leprosy may have potentially serious systemic inflammatory complications with a risk of neurological sequelae, functional limitation, and disability. Reactive states can affect up to 30–50% of patients with the multi-bacillary form. They may constitute a medical emergency when severe and rapid neurological damage develops. The standard treatment is prolonged corticosteroid therapy, often associated with important side effects, such as hyperglycemia, diabetes, osteoporosis, hypertension, cataracts, and immunosuppression, which can put patients at risk of serious infections, such as tuberculosis or strongyloidiasis (68).

**Figure 2:**
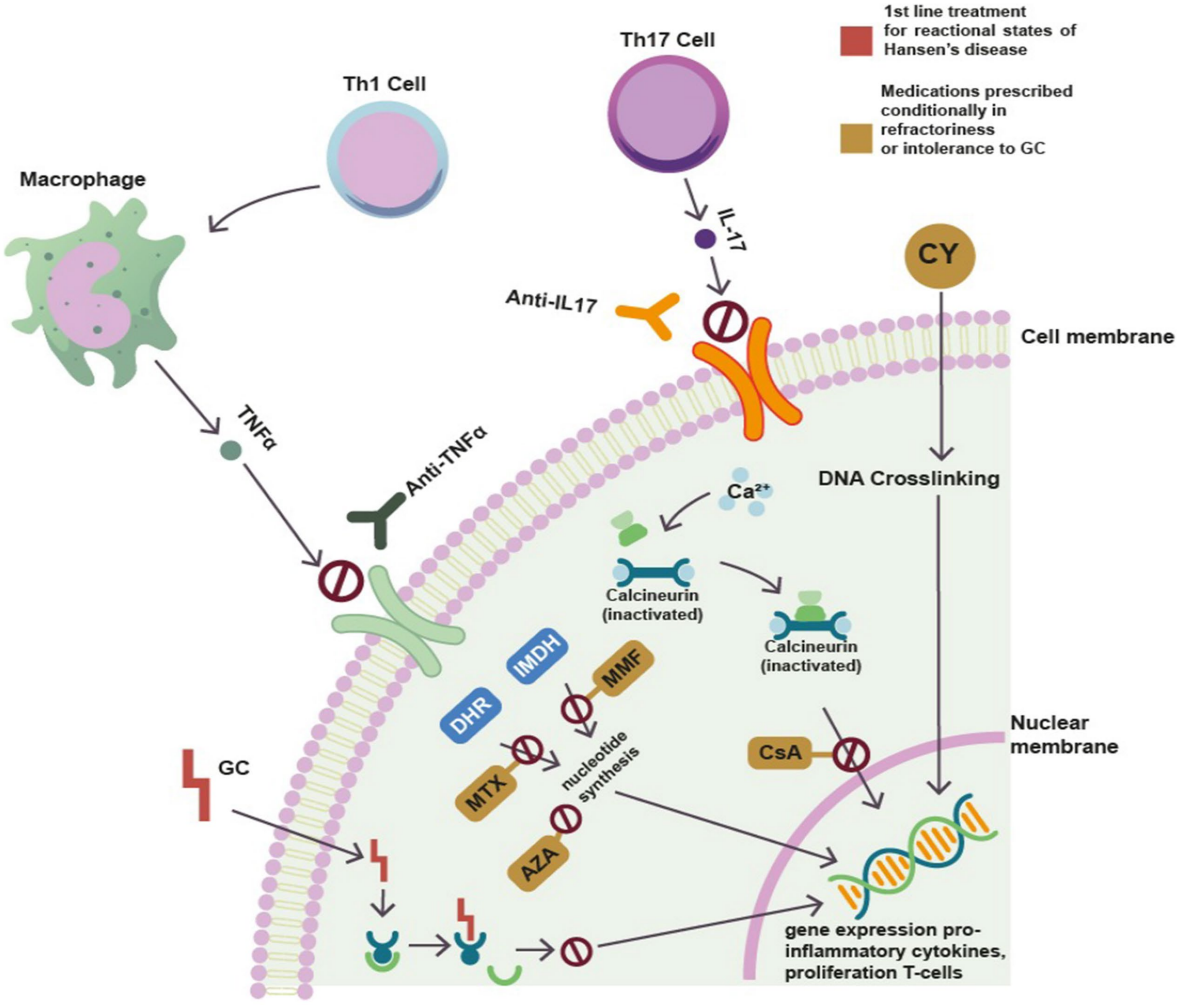
Synthetic and biological disease-modifying antirheumatic drugs and immunosuppressive agents used in the treatment of leprosy reactions and their action mechanisms. GC, glucocorticoid; MTX, methotrexate; AZA, azathioprine; MMF, mycophenolate mofetil; CsA, cyclosporine; CY, cyclophosphamide; anti-TNF, monoclonal antibodies against TNF alpha; anti-IL17, monoclonal antibodies against IL 17; IMDH, inosine monophosphate dehydrogenase; DHR, dihydrorhodamine. Modified from Diehl et al. (69).

Some immunosuppressive drugs used in rheumatology practice have been used in leprosy, such as corticosteroid sparing agents, to avoid these side effects and to facilitate the control of the signs and symptoms of the reactional states (Figure 2). Furthermore, up to 40% of patients may not respond to the corticosteroid regimen, and options such as high-dose thalidomide and clofazimine may be limited by significant adverse effects (70).

In the past, cyclophosphamide and antimalarials, especially chloroquine, were used as possible alternatives to control symptoms related to reactional states of leprosy, with reports of improvement in neuropathic symptoms, joint pain, and swelling. Over the years, they have been replaced by safer and more effective treatment options (71, 72).

Methotrexate, an antimetabolite of folate, offers some advantages that make it a good substitute or adjunctive treatment for corticosteroids. They have extensive experience with its use due to its favorable safety profile, easy dosing, and low cost. It seems effective in type 1 and 2 reactions that respond poorly to corticosteroids and thalidomide. However, the evidence on which its indication is based appears only in case reports and short clinical series (73, 74). High doses are generally not necessary, as the average dose of 15 mg per week has been recommended by most authors. The results of clinical trials that explore the role of methotrexate in the treatment of reactive states of leprosy are still awaited, including comparisons of regimens in monotherapy and in combination with other immunosuppressants (75, 76).

Azathioprine is an immunosuppressant that is metabolized to mercaptopurine, which inhibits the development of T cells. A study in leprosy patients with type 1 reactions that compared a combination of azathioprine and prednisolone versus prednisolone in monotherapy for 3 months revealed that the dose of corticosteroid needed for symptom control was less with the combined regimen (77).

Lockwood et al. followed 345 patients in a randomized study, comparing corticosteroid monotherapy with combined use with 50 mg of azathioprine in a fixed dose. They did not observe benefits in the assessment of the severity of the type 1 leprosy reaction with the combined therapy (78).

Adverse effects from azathioprine can occur in up to 15% of patients and include nausea, vomiting, mucous ulcers, and bone marrow suppression. The use of this drug in leprosy management remains controversial and restricted to refractory reactional states (79).

Mycophenolate mofetil is an immunosuppressant drug used in the treatment of immune-mediated rheumatic diseases such as SLE. Its effectiveness is mainly due to the induction of apoptosis in activated T cells, eliminating clones that respond to antigenic stimulation. Its use in leprosy reactional states as a steroid-sparing agent, although not routine, has been indicated as an alternative for cases refractory or intolerant to other therapies (79).

Banerjee et al. followed 20 patients with erythema nodosum leprosy (ENL) aged between 30 and 50 years, 16 of whom were multi-bacillary, unresponsive to standard treatment, and had contraindications to systemic corticosteroid therapy. Mycophenolate mofetil was used continuously at doses between 1 and 2 g per day. Most cases showed a significant reduction of lesions in 1 month, with maintenance of the medication for 4–6 months for complete remission of the condition. The authors concluded that this medication may be an important steroid-sparing agent for type II reactions, with rapid symptom control and remission lasting 6–8 months. Due to the scarcity of randomized clinical trials, the benefit of this medication remains uncertain (80).

There is increasing evidence that peripheral nerve dysfunction in leprosy is strongly dependent on neurotrophin activity. The most important neurotrophin in leprosy is the nerve growth factor (NGF), which is reduced during the course of leprosy due to the presence of autoantibodies against NGF. Levels of these autoantibodies are lowered by the immunomodulatory activity of cyclosporine (CsA), which primarily controls pain and improves motor function and sensitivity. Therefore, suppression of anti-NGF and regulation of NGF levels may be attractive targets for immunomodulatory treatment and control of leprosy neuroimmune reactions, making CsA a possible alternative in refractory leprosy cases (81).

De Sena et al. in an observational study with 67 patients, observed that anti-NGF antibodies are present in the serum of patients with leprosy and can influence the outcome of the neuritis and that CsA can be a useful drug in controlling nervous impairment and pain in patients with leprosy. Lambert, in a randomized clinical trial with 73 patients with type 1 reaction, concluded that CsA can be a safe second-line alternative drug for patients with T1R who are not responding or are experiencing adverse events related to corticosteroid therapy (82).

Alternatives to corticosteroids and thalidomide are needed for the treatment of T1R and T2R. Medications such as methotrexate, mycophenolate, azathioprine, and cyclosporine may represent alternatives with a good safety profile for refractory cases and in special populations, such as diabetics, in whom long-term use of corticosteroids is problematic. Randomized clinical trials and more extensive clinical experience will be essential to defining the role of each drug in leprosy.

## 10. Leprosy and immunobiologics therapy

The use of biologic immunosuppressants (monoclonal antibodies) to treat many rheumatic diseases is now widespread and growing. Some biologics—particularly tumor necrosis factor alpha inhibitors (iTNF)—were associated with a higher risk of latent tuberculosis reactivation and the development of atypical forms of the disease (83). Given that leprosy is also a mycobacterial disease, concerns naturally arise about the risk of Hansen’s disease among biologics users, although data are more scarce here.

Several case reports and case series have described new development, reactivation, worsening, or accelerated progression of leprosy, with or without leprosy reactions, in patients exposed to biologics (such as infliximab, adalimumab, etanercept, and abatacept) for the treatment of different inflammatory immune-mediated diseases, such as RA, ankylosing spondylitis, psoriatic arthritis, and psoriasis (84–89). Usually, the biologic was discontinued after the diagnosis, but in some cases, it was reinstituted after the completion of anti-leprosy multi-drug therapy. Leprosy reactions in these reported cases (usually type 1 reactions) most commonly arise after discontinuation of the biologic and initiation of specific therapy, which might reflect rapid restoration of immunity upon withdrawal of the biologic (84).

A systematic review and meta-analysis by Barroso et al. evaluated the occurrence of leprosy in patients under immunosuppression for immune-mediated inflammatory diseases. Twenty studies (24 cases of leprosy in total) were included; 95.2% of the cases occurred in patients with rheumatic diseases; 54.2% (*n* = 13) were on biologics (iTNF: 12 cases, tocilizumab: 1 case). The detection rate of leprosy among immunosuppressed patients varied greatly across different countries, from 0.13 (in the USA) to 116.18 (in Brazil) per 100,000 patients per year. The overall detection rate of leprosy in immunosuppressed patients with rheumatic diseases was 84 (95% CI: 0–266) cases per 100,000 patients per year. The authors pointed out that this rate was 30 times higher than the overall world detection rate in 2018, for the general population, and six times higher than reported in endemic countries such as Brazil. However, the biologic-specific risk for the development of leprosy was not reported (90).

Another systematic review, by Cogen et al. inquired specifically on whether the occurrence of clinical leprosy was increased in patients on biologics. Ten cases of leprosy diagnosed after initiation of an iTNF were assessed; three other cases of leprosy in patients on biologics were identified but not included for lack of detailed information. The authors postulated that iTNF could be a risk factor for developing or reactivating leprosy; this diagnosis should be considered, particularly in patients with skin lesions and arthritis (suggestive of rheumatic disease), before starting an iTNF biologic. Leprosy should also be considered in patients who develop worsening eruptions and neurological symptoms during treatment with iTNF (91).

Gomes et al. conducted a longitudinal study from 2014 to 2019 to evaluate the risk of leprosy in patients using immunosuppressants for dermatological and rheumatological diseases (*n* = 268, 46.3% exposed to biologics or JAK inhibitors at some point) compared to individuals not exposed to immunosuppression (*n* = 137). Ten cases of leprosy were identified during the follow-up, 90% (9/10) of which were under immunosuppression by the time of diagnosis, and 40% had been exposed to biologics (4/10). High degree of immunosuppression was a significant risk factor for leprosy development (HR = 7.9; 95% CI = 1.5–41). Of note, iTNF were associated with significantly lower risk of leprosy compared to corticosteroids (RR = 0.09, 95% CI = 0.02–0.4) (92).

In quite a different direction, there have also been reports and proposals on the treatment of leprosy manifestations with biologics (including infliximab, etanercept, adalimumab, rituximab, and secukinumab) for severe, refractory cases. Several case reports described successful control of leprosy reactions/manifestations such as erythema nodosum leprosum and neuritis, with good tolerability and apparent safety, in patients exposed to biologics for the treatment of other immune-mediated diseases (93), or for managing refractory leprosy manifestations themselves (94–97). In most cases, the biologic could be discontinued after complete resolution of leprosy reactions with no recrudescence or after a long symptom-free period.

A clinical trial (reported in abstract form) randomized 74 patients with type 1 reactions to receive secukinumab (anti-IL-17A) or prednisolone for 20 weeks. Improvements in skin signs, nerve function, and quality of life were similar in both groups. Recurrences of leprosy reactions (high in both groups: about 25%) occurred significantly earlier in patients on secukinumab, who needed 10% more additional prednisolone. Notwithstanding, serious adverse events were less frequent with secukinumab compared to prednisolone alone. The authors concluded that secukinumab could be a safe alternative in the management of leprosy reactions in patients not improving with or tolerating prednisolone (98).

The systematic review by Cogen et al. already mentioned, also evaluated the use of biologics in treating leprosy reactions. Four case reports of previously refractory erythema nodosum leprosum (ENL) successfully treated with infliximab or etanercept were identified, and a fifth case of persistent ENL responsive to infliximab was originally presented by the authors. They concluded that iTNF appear to be effective in some cases of refractory ENL (91).

Similarly, Gomes et al. in the aforementioned longitudinal study of patients with rheumatic and dermatological diseases, also noted that no participant under immunosuppression exhibited leprosy reactions. Moreover, during the follow-up, two cases showed improvement in leprosy manifestations after initiation of biologics: one case had improvement in neuritis while on rituximab (anti-CD20), and another one had type 1 reactions controlled under secukinumab (anti-IL-17A) (92). The authors concluded that immunosuppression by different mechanisms of action, including biologics, seemed effective in controlling leprosy reactions. However, the potentially deleterious effect of immunosuppressants on the cure of leprosy—with the theoretical risk of future reactivation—must always be considered.

## 11. Conclusion

Leprosy can mimic many different rheumatic conditions and must be investigated in cases with unexplained joint manifestations as these may be the first and only symptom presented, especially in endemic areas or travelers from endemic countries. A delayed diagnosis of leprosy may lead to irreversible structural joint damage. Immunosuppressive agents used in the treatment of immune-mediated rheumatic diseases can be conditionally prescribed in the management of refractory reactional conditions. On the other hand, patients with rheumatic diseases and a high degree of immunosuppression, especially those using corticosteroids and anti-TNFs, may be at greater risk of developing *M. leprae* infection.

## Author contributions

All authors listed have made a substantial, direct, and intellectual contribution to the work and approved it for publication.

## Conflict of interest

The authors declare that the research was conducted in the absence of any commercial or financial relationships that could be construed as a potential conflict of interest.

## Publisher’s note

All claims expressed in this article are solely those of the authors and do not necessarily represent those of their affiliated organizations, or those of the publisher, the editors and the reviewers. Any product that may be evaluated in this article, or claim that may be made by its manufacturer, is not guaranteed or endorsed by the publisher.
